# Predictors of Function, Activity, and Participation of Stroke Patients Undergoing Intensive Rehabilitation: A Multicenter Prospective Observational Study Protocol

**DOI:** 10.3389/fneur.2021.632672

**Published:** 2021-04-08

**Authors:** Bahia Hakiki, Anita Paperini, Chiara Castagnoli, Ines Hochleitner, Sonia Verdesca, Antonello Grippo, Maenia Scarpino, Antonio Maiorelli, Irene Eleonora Mosca, Paola Gemignani, Marco Borsotti, Maria Assunta Gabrielli, Emilia Salvadori, Anna Poggesi, Giulia Lucidi, Catiuscia Falsini, Monica Gentilini, Monica Martini, Maria Luisa Eliana Luisi, Barbara Biffi, Paolo Mainardi, Teresa Barretta, Silvia Pancani, Andrea Mannini, Silvia Campagnini, Silvia Bagnoli, Assunta Ingannato, Benedetta Nacmias, Claudio Macchi, Maria Chiara Carrozza, Francesca Cecchi

**Affiliations:** ^1^IRCCS Fondazione Don Carlo Gnocchi, Florence, Italy; ^2^NEUROFARBA Department, Neuroscience Section, University of Florence, Florence, Italy; ^3^Istituto di Biorobotica, Scuola Superiore Sant'Anna, Pisa, Italy; ^4^Department of Experimental and Clinical Medicine, University of Florence, Florence, Italy

**Keywords:** stroke, rehabilitation, functional recovery, biomarkers, neurophysiology

## Abstract

**Background:** The complex nature of stroke sequelae, the heterogeneity in rehabilitation pathways, and the lack of validated prediction models of rehabilitation outcomes challenge stroke rehabilitation quality assessment and clinical research. An integrated care pathway (ICP), defining a reproducible rehabilitation assessment and process, may provide a structured frame within investigated outcomes and individual predictors of response to treatment, including neurophysiological and neurogenetic biomarkers. Predictors may differ for different interventions, suggesting clues to personalize and optimize rehabilitation. To date, a large representative Italian cohort study focusing on individual variability of response to an evidence-based ICP is lacking, and predictors of individual response to rehabilitation are largely unexplored. This paper describes a multicenter study protocol to prospectively investigate outcomes and predictors of response to an evidence-based ICP in a large Italian cohort of stroke survivors undergoing post-acute inpatient rehabilitation.

**Methods:** All patients with diagnosis of ischemic or hemorrhagic stroke confirmed both by clinical and brain imaging evaluation, admitted to four intensive rehabilitation units (adopting the same stroke rehabilitation ICP) within 30 days from the acute event, aged 18+, and providing informed consent will be enrolled (expected sample: 270 patients). Measures will be taken at admission (T0), at discharge (T1), and at follow-up 6 months after a stroke (T2), including clinical data, nutritional, functional, neurological, and neuropsychological measures, electroencephalography and motor evoked potentials, and analysis of neurogenetic biomarkers.

**Statistics:** In addition to classical multivariate logistic regression analysis, advanced machine learning algorithms will be cross-validated to achieve data-driven prognosis prediction models.

**Discussion:** By identifying data-driven prognosis prediction models in stroke rehabilitation, this study might contribute to the development of patient-oriented therapy and to optimize rehabilitation outcomes.

**Clinical Trial Registration:** ClinicalTrials.gov, NCT03968627. https://www.clinicaltrials.gov/ct2/show/NCT03968627?term=Cecchi&cond=Stroke&draw=2&rank=2.

## Introduction

Stroke is one of the major causes of death and permanent disability in Western countries, with a growing impact on public health (1). Thrombolytic therapy represents a great progress in the treatment of the acute phase of cerebrovascular disease, but only a minority of patients are eligible and can actually receive it, and, on the whole, this improvement does not balance the steady increase of stroke prevalence, both for the greater longevity of the population and for the lower mortality in the acute phase. There is wide diversity in stroke severity and stroke patients (2); of those surviving a stroke, about 65% present disability and receive rehabilitation. However, deficits in activity and participation persist in about 30% of the survivors even after rehabilitation (3), and, to date, very little is known about long-term functional outcomes (4, 5). Stroke recovery is heterogeneous in its nature, but the intensity, quality, and timing of rehabilitation play a central role in the patients' recovery and in their reintegration into the community after a stroke (6). An interdisciplinary, multi-professional intensive rehabilitation approach tackling not only sensorimotor impairment but also all the possible associated problems, such as language, swallowing, sphincter and respiratory impairments as well as pain, depression, cognitive and/or communication disability, is highly recommended (6). As to physiotherapy, intensive exercise, consisting of increased repetitions and aerobic training, seems to optimize motor and functional outcome in stroke survivors with post-acute sensorimotor disability (7). However, in Italy, the implementation of interdisciplinary intensive rehabilitation for stroke survivors is highly diverse across the country. This is due to a remarkable interregional and intraregional heterogeneity in the rehabilitation pathways, leading to a high risk for inequalities and suboptimal care (8) as well as to major difficulties in quality assessment and benchmarking.

Moreover, the complex nature of stroke sequelae requires several assessment instruments to correctly quantify every residual symptom and adequately respond to the patients' needs during the acute, the post-acute, and the community-living stroke phase. Standardized assessment tools need to be easy to use and comprehensive of all the elements necessary to accurately address the great range of different rehabilitation needs (9).

In this context, the conduction of multicentric studies and clinical trials might be particularly difficult. Indeed a standardized, reproducible, and uniform outcome assessment, as much as a well-defined rehabilitation pathway, is necessary to investigate the effects of a single intervention during intensive interdisciplinary rehabilitation as well as for investigating individual predictors of response to treatment. Predictors may also differ for different interventions (10), suggesting clues to personalize rehabilitation and, possibly, improve rehabilitation outcomes (11). To date, a large representative cohort study focusing on individual variability of response to a standardized, evidence-based treatment is lacking (12), and predictive factors of individual response to treatment are still largely unexplored (13).

The significant inter-individual variability in the outcome of neurorehabilitation is related to the quality of medical and rehabilitation treatment in the different phases (14). However, a complex interaction of baseline health status (age and previous comorbidity) and physical/cognitive state, stroke subtype and severity as well as changes in brain structural architecture (15) is believed to largely influence stroke outcome. Furthermore, complications in the early-acute phase (16) also predict outcomes, for instance, post-stroke epilepsy (PSE) that has a high recurrence rate (30%) 1 year after the acute event (17) with approximately 50% of patients experiencing a recurrence of symptoms during a follow-up period of 47 months (18).

Recent studies have also highlighted the importance of some neurophysiological biomarkers, such as resting state electroencephalography (EEG) and motor evoked potentials (MEP), as predictors of motor recovery after a stroke (19). Finally, a recent line of research is focusing on genetic biomarkers since some genes, in particular, genetic variations of the brain-derived neurotrophic factor (BDNF) (20), catechol-O-methyl transferase polymorphism (21), and dopamine receptor (22), have been implicated in stroke recovery and prognosis (23).

Fondazione Don Carlo Gnocchi (FDG) is one of the largest Italian scientific rehabilitation and research institutions (SRRI) that have recently developed and implemented an evidence-based interdisciplinary integrated care pathway (ICP) for post-acute stroke inpatient rehabilitation (24). After a pilot study confirming feasibility and suggesting improved outcomes of the ICP compared to previous practice, this has been adopted in four intensive rehabilitation units (IRUs) in order to standardize the outcome definition and the process of care according to national and international stroke rehabilitation guidelines (6, 25).

This paper describes the background and methods of a multicenter study protocol to prospectively investigate outcomes and baseline predictors (including biomarkers) of function, activity, and participation of patients undergoing intensive interdisciplinary inpatient rehabilitation after a stroke. The purpose of the study will be to describe the influence of several clinical, functional, and psychosocial factors, neurophysiological patterns, and genetic polymorphisms on the recovery of body functions, activity, and participation of stroke survivors undergoing rehabilitation, both in the late-acute phase (discharged from IRUs) and in the chronic phase (6 months after a stroke).

## Materials and Methods

### Study Design and Setting

The study is a prospective, longitudinal, and observational cohort study including four IRUs of the FDG, located in two Italian regions: Tuscany and Liguria.

All patients admitted to the IRUs of the participating FDG centers (Florence, Massa, Fivizzano, and La Spezia) with a history of ischemic or hemorrhagic stroke within 30 days will be considered for eligibility for this study.

### Participants, Recruitment, and Data Collected

All patients admitted to the participating IRUs and fulfilling the following inclusion criteria will be assessed and treated according to the evidence-based stroke ICP and will be considered eligible for the study.

Inclusion criteria:

- First-ever or recurrent ischemic or hemorrhagic stroke
- Stroke diagnosis confirmed clinically and by brain imaging
- Acute event within 30 days
- Age 18+
- Written informed consent

Exclusion criteria:

- Transitory ischemic attack
- Patients with severe hemorrhagic or ischemic stroke (disorders of consciousness states and critical clinical care conditions) addressed to the severe brain injury high-complexity rehabilitation ward

All eligible patients will be asked to allow anonymous treatment of their data to participate in the study, and those who will sign the informed consent will be consecutively enrolled. For patients who were unable to provide or deny consent and without an available legal representative, the decision will be made by a specialist-in-charge who will take responsibility to act in the patient's best interest and will inform the patient's family and caregivers, according to the Florence university ethical committee indications.

Based on the average number of stroke patients referred to the four IRUs involved in the study in the previous years, it has been planned to enroll 270 patients (90 from Florence, 90 from La Spezia, and 90 from Massa and Fivizzano) in 12 months. The participants' enrollment will be based on a purposive sampling technique.

In order to address potential pitfalls related to a reduced recruitment of patients, specific actions have been considered, such as the realization of a detailed informed consent for the patients and offering a follow-up visit together with a full examination, and free blood exams, the results of which are directly provided to the medical practitioner.

The multi-professional staff (i.e., physiatrists, neurologists, physiotherapists, speech therapists, neurophysiologists, neuropsychologists, occupational therapists, nutritionist, and nurses) of each center will perform clinical and neurophysiological assessment by gathering clinical and neurophysiological data and structural indices of overall brain damage through brain imaging. At the coordinator center (Florence, Italy), a SRRI, a nutritional assessment and a blood draw will also be performed. A blood sample of each participant will be collected at T0 and T2 for the analysis of genetic markers that will be performed by the Neurogenetic Laboratory of the Careggi Teaching Hospital (Florence, Italy).

**Figure 1:**
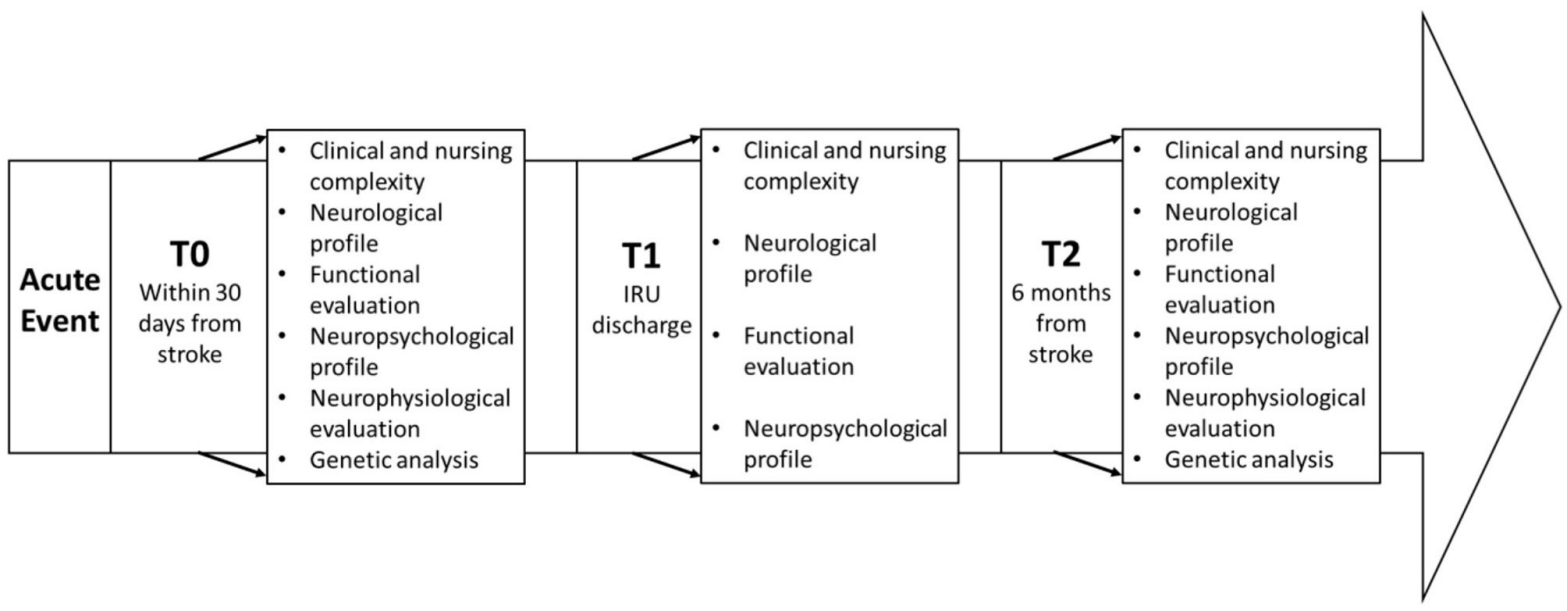
Study timeline.

Measures will be taken at (1) inpatient rehabilitation—admission (T0), (2) inpatient rehabilitation—discharge (T1), and (3) follow-up visit 6 months after the acute event (T2). The timeline of the study is illustrated in Figure 1.

### Clinical Evaluation

The clinical assessment will include a dataset of variables grouped into four categories:

- Clinical and nursing complexity (T0–T1 and T2) including the presence and severity of comorbidities, assessed through the Cumulative Illness Rating Scale, and the level of pain, assessed by the Numeric Rain Scale or the Pain Assessment in Advanced Dementia. Further markers of complexity include the presence of medical devices (e.g., tracheostomy, bladder catheter, nasogastric tube, etc.) and of pressure ulcers, incontinence, reduced vigilance, delirium, clinical instability, anemia, dialysis, and depression. Moreover, depression was assessed through the Hospital Anxiety Depression Scale or the Stroke Aphasia Depression Questionnaire. Additionally, dysphagia was assessed by the water swallow test and/or Functional Oral Intake Scale. Finally, malnutrition screening (MUST) will be performed at admission and weekly; the severity of malnutrition (GLIM) (26) will be defined at admission and discharge. Each patient will receive a personalized nutritional plan, tailored on their nutritional assessment, which included the evaluation of nutritional habits, administration route (oral or artificial), and laboratory exams (blood count, proteins, albumin, transferrin, ferritin, vitamins, minerals, CRP, TSH and cholesterol).

- Neurological profile including:
  - At admission (T0): stroke subtype fulfilling the Oxfordshire Community Stroke Project and Trial of Org 10172 in acute stroke treatment for ischemic strokes or intracerebral vs. subarachnoid for hemorrhagic stroke classifications and localization and extension of the brain damage as described by brain imaging, presence of clinical complications in the acute phase (e.g., late epileptic event, loss of consciousness, etc.), treatment received during the acute phase (thrombolytic therapy, neurosurgery, etc.), and procedural complications if these occurred.
  - At discharge (T1) and follow-up (T2): any neurological event occurring between the T0 and T1 and between T1 and T2 will be registered (late epileptic event, recurrence, etc.).

Overall neurological impairment will be assessed by the National Institutes of Health Stroke Scale that will be reassessed at each visit time.

- Functional evaluation including measures of impairment, disability, and participation.
  - Sensorimotor impairment will be assessed at all-time points by the trunk control test (TCT), Fulg–Meyer Assessment Scale (FMA), and modified Ashworth Scale.
  - Disability will be measured at all time points by the modified Barthel Index (mBI), the modified Rankin scale, the Scale of Disability in Communication (SDC) and the Functional Ambulation Categories (FAC), and the Short Physical Performance Battery.
    Participation will be assessed at T0 (anamnestic pre-stroke) and T2 (6 months from stroke) by the Frenchay Activity Index (FAI) and the Functional Walking Categories (FWC).

- Neuropsychological evaluation at T0 will include two tests of global cognitive screening, the Montreal Cognitive Assessment (MoCA) and the Oxford Cognitive Screen (OCS), followed by a minimal neuropsychological battery as detailed in Table 1. Two parallel versions and a third parallel version of the MoCA test will be administered, respectively, at T1 and at T2 as well as the same minimal neuropsychological battery administered at baseline.

**Table 1:** Administrated scales and evaluations.

**Area of competence**	**Evaluation tool**	**T0**		**T1**	**T2**
		**Anamnestic evaluation**	**Baseline evaluation**		
Clinical and nursing complexity	Cumulative Illness Rating Scale		X	X	X
	Markers of complexity		X	X	X
	Body mass index		X		
	Functional Oral Intake Scale/water swallow test		X		
	Numeric Rain Scale/pain assessment in advanced dementia		X	X	X
	Hospital Anxiety Depression Scale		X		
	Stroke Aphasia Depression Questionnaire		X		
	Presence of transitional adverse event			X	X
Neurological profile	National Institutes of Health Stroke Scale		X	X	X
	Trial of Org 10172 in Acute Stroke Treatment		X		
	Oxfordshire Community Stroke Project		X		
	Stroke localization and extension early clinical complications, thrombolysis/fibrinolysis		X		
	Presence of new neurological events			X	X
Functional evaluation	Trunk Control Test		X	X	X
	Fulg–Meyer Assessment Scale		X	X	X
	Short Physical Performance Battery		X	X	X
	Modified Barthel Index		X	X	X
	Modified Rankin scale	X	X	X	X
	Scale of disability in communication		X	X	X
	Functional ambulation categories		X	X	X
	Medical Research Council Scale		X	X	X
	Activity of daily living disabilities: Frenchay Activity Index	X			X
	Activity of daily living disabilities: functional walking categories	X			X
Neuropsychological evaluation	Montreal Cognitive Assessment		X	X	X
	Oxford Cognitive Screening		X		X
	Babcock story recall test		X		X
	Digit span forward and backward		X		X
	Symbol Digit Modalities Test		X		X
	Stroop Test		X		X
	Token and naming subtests of the Aachen Aphasia Test		X		X
	Fluff test		X		X
	Ideomotor apraxia test		X		X
Neurophysiological evaluation	Electroencephalography		X		X
	Motor evoked potential		X		X
Genetic markers	Brain-derived neurotrophic factor		X		X

All assessment tools are shown in Table 1, and their references can be found in the supplementary material (Supplementary Table 1).

### Neurophysiological Assessment (T0–T2)

#### Electroencephalography

An EEG of 20 min of wakefulness will be performed using Galileo NT (EBNEURO) with 32 channels through an EEG prewired Headcap19 recording electrode, one ground electrode, and a reference positioned according to the 10–20 International System. The signal will be sampled at 128 Hz, with sensitivity set at 7 uV/mm, and then filtered (1.6–30 Hz). The examination will be carried out on a chair or on a wheelchair on the basis of the patient's clinical conditions. During the 20-min recording period, patient reactivity will be assessed through active or passive eyes' opening and closing depending on the degree of patient collaboration. Hyperventilation will not be performed as most patients have contraindications arising from age and cardiac and respiratory problems associated with cerebrovascular insult. Electroencephalographic abnormalities will be classified according to the American Clinical Neurophysiology Society's standardized critical care EEG terminology (27, 28). In particular, epileptiform abnormalities will be classified as follows: (1) interictal epileptiform activity, (2) periodic discharges, and (3) electrographic seizures.

Reliability and quality control among the repeated measurements in the two centers (Florence and La Spezia) will be guaranteed by the use of the same instrumentation, the same EEG technicians performing the examination, and the same experimenters conducting the signal classification.

#### Motor Evoked Potentials

Transcranial magnetic stimulation (TMS) will be performed only in the center of Florence according to the standard criteria of the International Federation of Clinical Neurophysiology (29). Five consecutive responses in a 100-ms post-stimulus period will be analyzed. The size of MEP induced by TMS will be expressed as MEP/CMAP amplitude ratio, where MEP is the average of 5 consecutive peak-to-peak (most negative to the most positive peak) responses amplitude and CMAP is the Compound Muscle Action Potential of target muscle, evoked by supramaximal electrical peripheral nerve stimulation (ulnar nerve at the wrist for Abductor Digiti Minimi, ADM, Peroneal Nerve at Knee for Tibiali anterior, TA). The procedure will be performed for both upper limbs in all subjects.

The exclusion criteria are (1) Medical Research Council grade ≥4 (patients were able to perform a movement in all amplitudes even against minimal resistance by the examiner), (2) cognitive deficit hampering the patient's collaboration, (3) presence of a pacemaker, and (4) presence of physical limitations (e.g., bandages or plasters) that prevent the evaluation of peripheral muscular response through the CMAP.

The examination will be conducted with the Medelec Synergy electromyograph (Natus Europe) associated with the MagVenture MagPro Compact magnetic stimulator equipped with a circular coil.

Disposable surface electrodes (Bionen Florence, Italy) will be placed on the muscle under examination.

Once the motor response of supramaximal amplitude will be obtained, the magnetic stimulus will be delivered. A circular coil will be used to map the area of interest corresponding to the target muscles studied: for the TA, given the cortical representation, the coil will be positioned slightly ipsilateral to the side under examination in correspondence to Cz, while for the ADM, the coil will be positioned contralaterally taking Cz as reference. By asking the patient to perform a minimal contraction, at least five responses will be obtained and averaged in order to obtain reliable measures. A sixth stimulus will be delivered in a resting situation for interside comparison in those cases where the patient will be unable to activate the affected side. A stimulus will be provided at the paraspinal level only in the assessment of the upper limbs due to the increased accessibility of the stimulation site.

The MEP will be classified as follows:

- Normal
- Pathological if the MEP will be recordable but not within the reference values for amplitude and/or absolute latencies
- Absent if no response higher than 50 μV could be obtained after 5 stimuli at 100% intensity

The study foresees a further evaluation of the neurophysiological parameters during follow-up (T2), which corresponds to 6 months after a stroke.

### Analysis of Genetic Markers (T0–T2)

For the subgroup of patients in the Florence Intensive Rehabilitation Unit who will sign a further dedicated informed consent, a blood sample, along with the routine blood draw at T0 and T2, will be frozen and sent to the University Hospital Neurogenetic Lab. The study will focus on the polymorphism analysis of BDNF (T0); furthermore, the epigenetic analysis of the BDNF will be carried out by the evaluation of the methylation levels of the BDNF gene promoter. The DNA of the subjects will be extracted from peripheral blood using an automatic standardized method (QIAcube, QIAGEN). BDNF Val66Met polymorphism analysis (rs6265) will be performed by PCR amplification of the gene region containing the variant using the following primers—forward: 5′-ACT CTG GAG AGC GTG AAT GG-3′, reverse: 5′-TCCAGG GTG ATG CTC AGT AGT-3′.

Subsequently, high-resolution melting analysis will be carried out to identify individual variations of bases present in the amplified region. The melting curve generated by the analysis will be different for each genotype of polymorphism. Three control genotypes previously identified through sequencing will be used as references (310 ABI PRISM Genetic Analyzer, Applied Biosystem). If the promoter of the gene is found to be hyper-methylated, there will be a suppression of protein expression levels, resulting in a reduction in protein function. The epigenetic changes will be evaluated at two different times, at the patient's entry (T0) and at follow-up (T2), to study the possible influence of the treatment on the methylation levels of the BDNF gene.

The genetic polymorphism data will be compared to sex- and age-matched controls of the DNA bank of the Neurogenetic Laboratory.

### Rehabilitation

**Figure 2:**
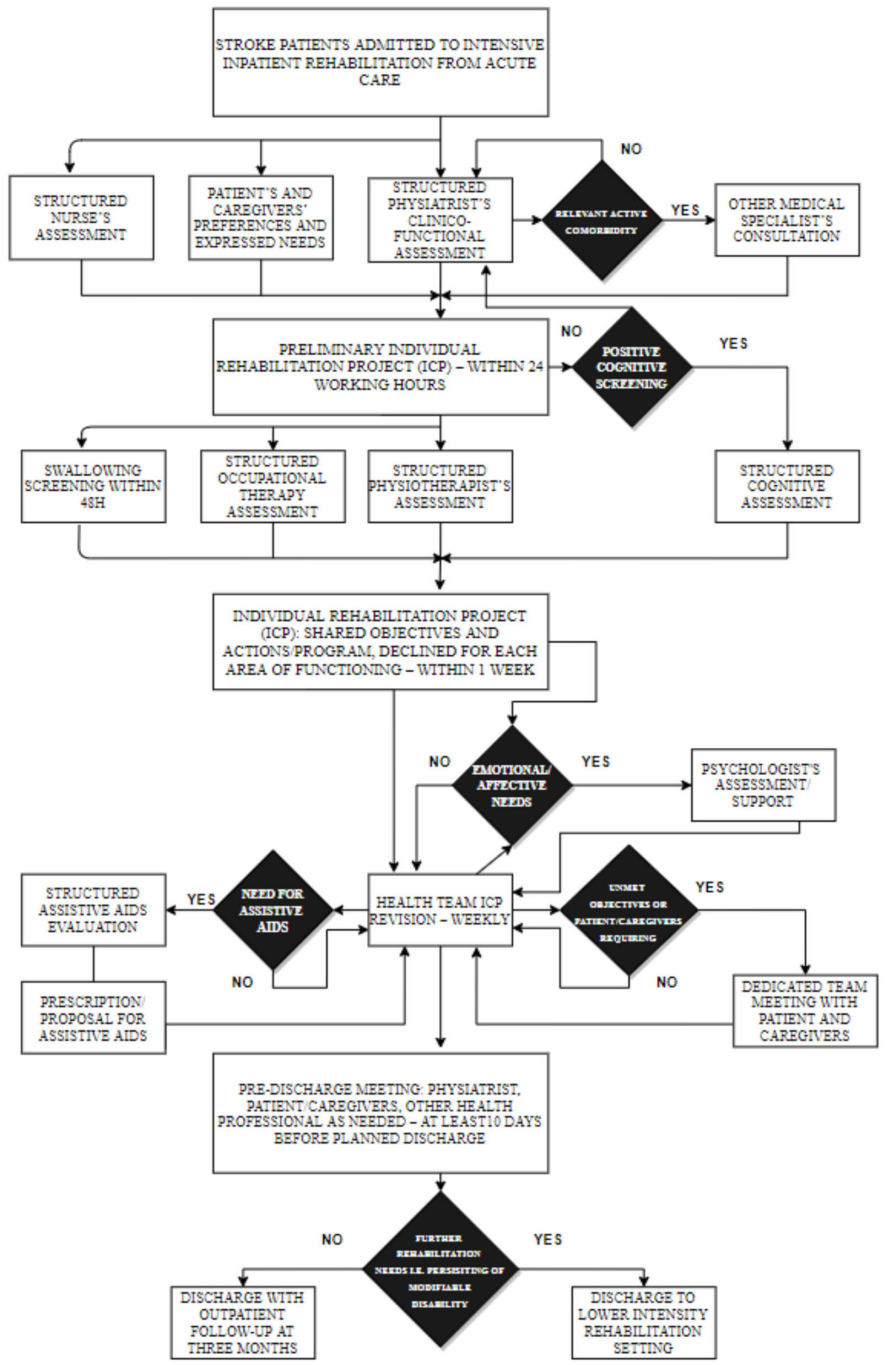
Rehabilitation treatment.

The rehabilitation intervention is defined in an ICP based on the *American Heart Association/American Stroke Association* guidelines and on the ICF model (6, 24), and detailed description and discussion can be found elsewhere (24). Synthetically, the standardized rehabilitation assessment and process of care provide, according to the national requirements, at least 3 h per day of specific rehabilitation including physiotherapy, neuropsychological therapy, speech and dysphagia therapy, and occupational therapy, in addition to the assessment and training in the use of aids, based on systematic screening at admission, systematic weekly team revisions of individual rehabilitation plan, and emerging needs any time during the rehabilitation stay. When indicated, psychological support to patient and or family is also provided. Physiotherapy may also include robotic rehabilitation of the upper limb according to the individual rehabilitation plan defined by the interdisciplinary team. Specific rehabilitative interventions are summarized in Figure 2.

### Outcome Measures

The primary outcome is improvement of functional ability that will be evaluated based on the change in the activities of daily living as assessed by the mBI, between T0 and T1, and between T0 and T2.

Secondary outcomes include cognitive recovery that will be evaluated by comparing the performances in the parallel versions of the MoCA, administered at all time points, and recovery of participation at 6 months, compared to pre-stroke data, according to the FAI and the FWC.

Other selected rehabilitation outcomes will include length of stay, adverse outcomes (post-stroke epilepsy, deaths, or discharge to acute care hospital), functional and clinical indicators recorded at admission and discharge for changes in sensory–motor impairment (FMA), SDC, TCT, and ambulation (FAC). Changes in markers of complexity will also be considered. Follow-up outcomes will include all of the above, EEG and MEP and BDNF epigenetics, intended as biomarkers of neuroplasticity. All demographic, clinical, functional, neuropsychological, and neurogenetic baseline variables will be analyzed as possible predictors/biomarkers of rehabilitation outcomes.

### Data Collection, Management, and Analysis

Data collection will be carried anonymously on REDCap, an online-based software for the design of databases. This will allow one to control the quality in data collection to reduce the number of missing data and then achieve a robust basis for the analyses.

Data will be secured by private credentials, saving the correspondences between names and identification codes on a separate document that will be destroyed at the end of the study.

Statistical analyses will be performed using SPSS 26.0 software (IBM SPSS, Chicago, Illinois, USA). Descriptive statistics of variables belonging to the four clinical assessment categories (clinical and nursing complexity, neurological profile, functional evaluation, and neurocognitive profile, neurophysiological parameters, and methylation levels of the BDNF gene) will be provided for each time point. The statistics will include absolute counts and relative frequencies for categorical and dichotomous variables, while mean and standard deviation or median and interquartile range will be according to the parametric or non-parametric distribution of the investigated numeric variables. Violation of the parametric assumption will be ascertained using the Shapiro–Wilk test.

Variables recorded at two time points will be compared using paired *t*-test or a Wilcoxon signed rank test as appropriate for the normality of the data; for numeric scales, the McNemar test for paired categorical or dichotomous data will be used instead. For those variables evaluated at all time points, differences over time will be ascertained using a repeated-measures ANOVA or Friedman test for continuous variables and Cochran's *Q* test for dichotomous variables, and Bonferroni and Sheffé corrections will be applied to multiple comparisons. In all the above-mentioned analyses, *p* value <0.05 will be considered as level of statistical significance.

In the second step of the analysis, four classes will be identified based on the mBI score: functional ability (FA) class 1: mBI <25, corresponding to a severe impairment; FA class 2: mBI between 25 and 50, corresponding to a moderate impairment; FA class 3: mBI between 50 and 75, corresponding to a mild impairment; and FA Class 4: mBI >75, corresponding to no impairment. Patients having missing values for the mBI will be excluded from further analyses.

Two groups will be then created based upon the FA class assessed at different time points (“improved FA” and “not improved FA”). Those variables identified through univariate analysis as recovery predictors will be grouped in five categories: clinical/nursing complexity (including anagraphical data), neurological profile, functional factors, cognitive factors, neurophysiological parameters, and genetic profile. For each category, a multiple logistic regression analysis will be conducted to investigate whether there is a relationship with improved FA. The analysis will be then repeated, including variables from different categories that showed a significant association with the presence of a higher FA class at discharge or follow-up.

In addition to the classical multivariate logistic regression analysis, advanced machine learning algorithms will be cross-validated to achieve data-driven prognosis prediction models.

Classical solutions such as linear and logistic regression will be compared with solutions based on support vector machines, random forests, or multi-layer perceptrons and also with “deep” artificial neural networks (convolutional neural networks, recurrent neural networks, and ensemble learning solutions).

The performances of different algorithms in predicting recovery outcomes will be compared in terms of accuracy, F1 score, root mean square error, and determination coefficient. The effect of hyperparameter tuning and automatic features selection strategies will also be tested by nested cross-validation, with the final aim to quantify the solution generalization capability when applied to new patients who were not included in model definition and training.

## Discussion

The past decade has seen great progress in the treatment of cerebrovascular diseases in the acute phase. However, stroke remains a catastrophic event of huge public health consequence, causing five million deaths each year and an increasing number of persons surviving with chronic disability worldwide (30). Recovery from stroke is a complex process that is achieved through a combination of spontaneous learning-dependent steps, including restoration (of the functionality of neural damaged tissue), substitution (of partly spared neural pathways to relearn lost functions), and compensation (leading to improve the disparity between impaired skills and the environmental requirements). Rehabilitation is recognized to effectively reduce post-stroke disability burden (31), but stroke rehabilitation presents specific challenges for research, especially in the definition of outcome assessment and in the customization of rehabilitation strategies. To allow the evaluation and comparison of rehabilitation outcomes on large populations of stroke patients (32) and to investigate predictors/biomarkers of rehabilitation outcomes, it is mandatory that evidence-based rehabilitation assessment and care processes are clearly defined and comparable, yet systems of care, approaches to stroke rehabilitation delivery, and outcome assessment as well as resources for stroke care and rehabilitation are still extremely variable across geographic regions worldwide (33).

In Italy, stroke rehabilitation recommendations make general reference to national and international guidelines (34), but their operational definition to protocols and pathways is very heterogeneous and significantly affected by the different standards applied at the regional and even the local level, creating a high risk for suboptimal care (8). To date, there is no standard quality assessment for the benchmarking of different rehabilitation facilities and strategies (9).

Furthermore, the customization of rehabilitation treatment according to individual predictors and relatively novel biomarkers of rehabilitation outcome would provide a ratio attempting in the optimization of the rehabilitation outcomes. However, this research is strongly limited by the lack of standardization of the applied rehabilitation pathway and of adequately validated prediction models.

Indeed optimization of rehabilitation strategies is yet largely limited by the restricted knowledge existing on biomarkers of rehabilitation outcome: thus, besides the clinical–functional component, the present study will also investigate neurophysiological and genetic parameters as possible biomarkers of functional outcome.

EEG, in addition to the identification of epileptic abnormalities as possible predictive index of PSE occurrence, could allow data for quantitative analysis (connettoma) for the eventual prediction of PSE onset. PSE has been identified as a significant clinical issue in stroke survivors, with an incidence rate of around 7% (35). It has also been associated with a poor prognosis and increased mortality in post-stroke survivors (36).

As to motor recovery, it will also be verified whether and how the combination of the collected neurophysiological measures (EEG and MEP) could contribute to motor prognosis. Recent studies have emphasized the importance of some neurophysiological markers as possible predictors of motor recovery after a stroke (19). In particular, an ipsilesional loss of power in the alpha frequency band and an increase in the delta frequency band in the EEG detected within 2 weeks of stroke are linked to a poor outcome (37), and quantitative EEG biomarkers might predict motor recovery within 3 weeks of stroke symptom onset (38). As to TMS that evaluate the integrity of human motor pathways (39), corticospinal excitability measured by TMS has been identified as a possible biomarker of upper limb motor function in patients with stroke (11, 19). MEP of the extensor digitorum communis amplitudes within the first 4 weeks after stroke also seems to be associated with a higher score in the FMA-UE26 (40). The assessment of residual central and peripheral neural motor circuitry complementing TMS analysis with motor nerve conduction exams might increase the prognostic accuracy for motor and functional outcome in patients with stroke.

As for neurogenetic potential biomarkers, we focused on the epigenetics of the single-nucleotide polymorphism of the BDNF gene that has been reported to impact neuroplasticity and motor learning in humans. BDNF is known to play a pivotal role in synaptic plasticity and enhanced neurogenesis in adults (41), and BDNF polymorphism is also known to be associated with post-stroke motor learning in adults. Interestingly, the effect of BDNF polymorphism on motor learning is likely influenced by the extent to which motor practice engages neuroplasticity and therefore by the rehabilitative approach. The *Met* allele carriers were shown to perform worse compared with *Val/Val* phenotype (42) and showed a reduction in the magnitude of motor improvement with therapy (43). Kim et al. (44) also reported that BDNF *Val66Met* polymorphism affected the degree of corticospinal degeneration. More recently, Oh et al. (45) showed that the degree of swallowing recovery after a stroke could be influenced by a polymorphism in BDNF *rs6265*.

An increasing number of studies focus on the use of demographic, clinical, and neurological variables to predict functional outcomes after a stroke, and several studies use automatic tools to make predictions rather than simply providing multivariable regression equations. However, the use of these tools to predict discharge outcomes (46, 47) could represent a risk in clinical practice of justifying an early discharge once a specific level of function has been reached. Therefore, the objectives of the use of prediction tools need to be carefully addressed as part of their implementation to ensure that they are used to aid rehabilitation planning and goal setting rather than as a means of restricting access to services (48). Concretely speaking, previous research aimed at the automatic prediction of the probability of clinical binary outcomes, such as independent walking (46), performing a percutaneous endoscopic gastrostomy, and the probability of achieving at least one specific clinical score for independence (47) or upper limb activity (49). Other crucial aspects to be discussed from the existing predictive models in stroke populations is the overlooked necessity of an external validation of computational solutions and the exploration of predictors independently on the scales in use. Concerning the former point, the so-called overfitting of methods is a common issue of machine learning methods applied to clinical trials. Indeed the identification of predictive factors for a better rehabilitation outcome after stroke should be targeted to a specific population, but at the same time a performant predictive model should be able to generalize and extend its predictive power to new data (47). For what concerns the choice of predictor variables, many studies report as limitations a constrained choice of the variables used as predictors, resulting in an omission of potentially more relevant features for the selected outcome (50). The extensive assessment provided in this prospective study will allow a more detailed description of the population before and after the rehabilitation treatment and the possibility to exploit an automatically or statistically driven feature selection for the predictive models.

In the present work, the outcome measures have been chosen to reflect, as realistically as possible, the functions, activity, and participation of the patient, and the possible predictors identified will be used to better tailor the rehabilitation pathway for each individual patient. The study also aims to identify clinical and instrumental markers predicting different outcome categories in different time points (at discharge and at 6 months after the acute event), which may be highly relevant both to patients and clinicians. Thus, a comprehensive protocol for the stroke population will be generated and tested to be potentially applied in the clinical practice of many centers. The applicability of this protocol is also supported by the consideration that, with the exception of the neurogenetic assessment, all the clinical and instrumental variables selected in this study are easily exportable to other clinical realities because of their low cost and high feasibility.

In conclusion, the present study plans to identify reliable predictive factors of specific functional, cognitive, and participation domains in the late-acute and the chronic phase after stroke by exploring the complex interaction between clinical–functional variables, genetic markers, and changes in brain structural architecture using neurophysiological evaluations. In addition to the classical multivariate logistic regression analysis, advanced machine learning algorithms will be cross-validated to achieve data-driven prognosis prediction models. The final aim of these models is to develop patient-oriented therapy, addressing personalized treatment plans that could be recommended and translated in routine clinical practice for optimizing care management.

## Ethics Statement

The studies involving human participants were reviewed and approved by Ethics Committees (Florence: 14513, La Spezia: 294/2019; Massa e Fivizzano: 68013/2019). The patients/participants provided their written informed consent to participate in this study.

## Author Contributions

BH, CM, and FC contributed to the general study design and draft of the whole manuscript. GL contributed to neurological and neurogenetic assessment protocol design and draft of the whole manuscript. APa, CC, IH, and SV contributed to functional assessment protocol design. APo, CF, and MM contributed to neurological assessment protocol design. IM, ES, MB, and PG contributed to neuropsychological assessment protocol design. AG, MS, and AM contributed to neurophysiological assessment protocol design. ML and BB contributed to nutritional assessment protocol design. BN and SB contributed to neurogenetic assessment protocol design. SP, AM, and SC contributed to statistical analysis design. All authors contributed to the article and approved the submitted version.

## Conflict of Interest

The authors declare that the research was conducted in the absence of any commercial or financial relationships that could be construed as a potential conflict of interest.

## Abbreviations

PSE: post-stroke epilepsy
EEG: electroencephalography
MEP: motor evoked potential
BDNF: brain-derived neurotrophic factor
FDG: Fondazione don Carlo Gnocchi
SRRI: scientific rehabilitation and research institution
ICP: integrated care pathway
IRUs: intensive rehabilitation units
TCT: Trunk Control Test
MUST: Malnutrition Universal Screening Tool
FMA: Fulg–Meyer Assessment scale
ADLs: activities of daily living
mBI: modified Barthel Index
FAC: functional ambulation categories
FAI: Frenchay Activity Index
FWC: functional walking categories
MoCA: Montreal Cognitive Assessment
OCS: Oxford Cognitive Screening
TAM: tibialis anterior muscles
CMAP: compound muscle action potential
TMS: transcranial magnetic stimulation
FA: functional ability
PMIC: Protocollo di Minima per l'Ictus
MMSE: Mini-Mental State Examination
MI: motor index.
