# Supplementary material for: Predictors of Function, Activity, and Participation of Stroke Patients Undergoing Intensive Rehabilitation: A Multicenter Prospective Observational Study Protocol
Source: Front Neurol. 2021 Apr 8;12:632672. doi: 10.3389/fneur.2021.632672 (PMC8060493; doi:10.3389/fneur.2021.632672)
Supplement: Supplementary file 2 [file Data_Sheet_2.pdf]

Table 1: Supplementary material, Evaluation tools references

Area of competence	*Evaluation tool*	*Acronym*	*Reference*
Clinical and nursing complexity	Cumulative Illness Rating Scale	CIRS	Linn BS, Linn MW, Gurel L. CUMULATIVE ILLNESS RATING SCALE. Journal of the American Geriatrics Soci-ety. maggio 1968;16(5):622–6.
	Markers of complexity	/	Banchero A, Baratto L, Bellelli G, Bellia C, Bernardini B, Bressi M, et al. IPER2: Indicatori di processo e di Esito in Riabilitazione. :76.
	Functional Oral Intake Scale/water swallow test	FOIS	Crary MA, Mann GD, Groher ME. Initial psychometric assessment of a functional oral intake scale for dysphagia in stroke patients. Arch Phys Med Rehabil. 2005 Aug;86(8):1516-20. doi: 10.1016/j.apmr.2004.11.049. PMID: 16084801.
	Numeric Rain Scale/pain assessment in advanced dementia	NRS<br><br>PAINAD	High AS, Macgregor AJ, Tomlinson GE, Salkouskis PM. A gnathodynanometer as an objective means of pain as-sessment following wisdom tooth removal. British Journal of Oral and Maxillofacial Surgery. agosto 1988;26(4):284–91.<br><br>Costardi D, Rozzini L, Costanzi C, Ghianda D, Franzoni S, Padovani A, et al. The Italian version of the pain as-sessment in advanced dementia (PAINAD) scale. Archives of Gerontology and Geriatrics. marzo 2007;44(2):175–80.
	Hospital Anxiety Depression Scale	HADS	Snaith RP. The Hospital Anxiety And Depression Scale. Health Qual Life Outcomes. 2003;1:29. Published 2003 Aug 1. doi:10.1186/1477-7525-1-29
	Stroke Aphasia Depression Questionnaire	SADQ-10	Lincoln, Nadina & Sutcliffe, L.M. & Unsworth, G.. (2000). Validation of the Stroke Aphasic Depression Questionnaire (SADQ) for use with patients in hospital. Clin Neuropsychol Assess. 1. 88-96.
Neurological profile	National Institutes of Health Stroke Scale	NIHSS	Goldstein LB, Samsa GP. Reliability of the National Institutes of Health Stroke Scale: Extension to Non-Neurologists in the Context of a Clinical Trial. Stroke. febbraio 1997;28(2):307–10.
	Trial of Organization in Acute Stroke Treatment	TOAST	Adams HP, Bendixen BH, Kappelle LJ, Biller J, Love BB, Gordon DL, et al. Classification of subtype of acute is-chemic stroke. Definitions for use in a multicenter clinical trial. TOAST. Trial of Org 10172 in Acute Stroke Treatment. Stroke. gennaio 1993;24(1):35–41.
	Oxfordshire Community Stroke Project	OCSP	Anderson CS, Taylor BV, Hankey GJ, Stewart-Wynne EG, Jamrozik KD. Validation of a clinical classification for subtypes of acute cerebral infarction. Journal of Neurology, Neurosurgery & Psychiatry. 1 ottobre 1994;57(10):1173–9.
Functional evaluation	Trunk Control Test	TCT	Franchignoni FP, Tesio L, Ricupero C, Martino MT. Trunk Control Test as an Early Predictor of Stroke Rehabili-tation Outcome. Stroke. luglio 1997;28(7):1382–5.
	Fulg-Meyer Assessment scale	FMA	Cecchi F, Carrabba C, Bertolucci F, Castagnoli C, Falsini C, Gnetti B, et al. Transcultural translation and valida-tion of Fugl–Meyer assessment to Italian. Disability and Rehabilitation. 1 maggio 2020;1–6.

	Short Physical Performance Battery	SPPB	Guralnik JM, Simonsick EM, Ferrucci L, Glynn RJ, Berkman LF, Blazer DG, et al. A Short Physical Performance Battery Assessing Lower Extremity Function: Association With Self-Reported Disability and Prediction of Mor-tality and Nursing Home Admission. Journal of Gerontology. 1 marzo 1994;49(2):M85–94.
	Modified Barthel Index	MBI	Shah S, Vanclay F, Cooper B. Improving the sensitivity of the Barthel Index for stroke rehabilitation. Journal of Clinical Epidemiology. gennaio 1989;42(8):703–9.
	Modified Rankin scale	MRS	Quinn TJ, Dawson J, Walters MR, Lees KR. Exploring the Reliability of the Modified Rankin Scale. Stroke. marzo 2009;40(3):762–6.
	Scale of disability in communication	SDC	Scale of disabilty in communication Meinecke C, Cappadonia C, Bernardini B. Validazione di una semplice scala di impairment comunicativo nell'anziano. In: XXIII Congresso Nazionale SIMFER Valutazione e qualità dell'assistenza in Medicina Riabilitativa. Ancona; 1995. p. 56–7.
	Functional Ambulation Categories	FAC	Geyh S, Kurt T, Brockow T, Cieza A, Ewert T, Omar Z, et al. Identifying the concepts contained in outcome measures of clinical trials on stroke using the international classification of functioning, disability and health as a reference. Journal of Rehabilitation Medicine. 1 agosto 2004;36(0):56–62.
	Medical Research Council scale	MRC	Medical Research Council of the United Kingdom. Palo Alto, Calif: Pedragon House; 1978.
	Activity of Daily Living disabilities: Frenchay Activity Index	FAI	Monteiro M, Maso I, Sasaki AC, Barreto Neto N, Oliveira Filho J, Pinto EB. Validation of the Frenchay activity index on stroke victims. Arq Neuro-Psiquiatr. marzo 2017;75(3):167–71
	Activity of Daily Living disabilities: Functional Walking Categories	FWC	Perry J, Garrett M, Gronley JK, Mulroy SJ. Classification of Walking Handicap in the Stroke Population. Stroke. giugno 1995;26(6):982–9.
Neuropsychological evaluation	Montreal Cognitive Assessment	MoCA	Santangelo G, Siciliano M, Pedone R, Vitale C, Falco F, Bisogno R, et al. Normative data for the Montreal Cogni-tive Assessment in an Italian population sample. Neurol Sci. aprile 2015;36(4):585–91.
	Oxford Cognitive Screening	OCS	the Italian OCS Group, Mancuso M, Varalta V, Sardella L, Capitani D, Zoccolotti P, et al. Italian normative data for a stroke specific cognitive screening tool: the Oxford Cognitive Screen (OCS). Neurol Sci. ottobre 2016;37(10):1713–21.
